# Designing and Implementing a Customized Questionnaire to Assess the Attitude of Patients with Diabetes

**DOI:** 10.3390/healthcare13070815

**Published:** 2025-04-03

**Authors:** Angela Repanovici, Ileana Pantea, Nadinne Alexandra Roman

**Affiliations:** 1 Faculty of Product Design and Environment, Transilvania University of Brasov, 500036 Brasov, Romania; 2 Faculty of Medicine, Transilvania University of Brasov, 500036 Brasov, Romania

**Keywords:** diabetes mellitus, dietary influence, questionnaire, exploratory factor analysis, structural equation modeling

## Abstract

**Background/Objectives**: Diabetes mellitus presents significant management challenges, requiring comprehensive glycemic control, patient education, self-management, and routine monitoring. The study aims to evaluate existing tools and develop a customized questionnaire to investigate the multifaceted impact of diabetes mellitus on patients’ lives through a novel questionnaire. **Methods**: Utilizing Survey Monkey, we efficiently collected data from 150 diabetic patients during annual evaluations over five months (March 2024–July 2024). The sample included 88 men (58.67%) and 62 women (41.33%), with a notable representation of participants having a family history of diabetes (63.42%) and varying levels of education (20% with higher education). Statistical analyses were conducted using IBM SPSS (Version 20.0), and structural equation modeling (SEM) through Amos, including exploratory factor analysis (EFA) and confirmatory factor analysis (CFA) to validate the instrument and assess its psychometric properties. **Results**: The questionnaire targets four critical domains: the role of physical activity in diabetes management, the effects of diabetes on social relationships, the emotional status of diabetic patients, and the influence of diet on metabolic control. **Conclusions**: The findings provide valuable insights into patient attitudes toward diabetes management, emphasizing the importance of physical activity, social dynamics, emotional well-being, and dietary practices in improving health outcomes for individuals with diabetes.

## 1. Introduction

Diabetes mellitus presents a considerable challenge in management and care, requiring a comprehensive approach that includes glycemic control, patient education, self-management, and routine monitoring. Multidisciplinary interventions often lead to better outcomes by addressing the disease and its associated risk factors [1]. Effective diabetes management reduces the risk of complications and improves overall quality of life, underscoring the importance of continued research and adaptation of strategies [2].

Previous studies highlight that patient knowledge significantly influences diabetes self-care practices. Knowledge about diabetes is a key factor in enabling effective self-care practices. When individuals understand their condition, they are equipped with the necessary skills to manage their health and make informed decisions [3]. Knowledge impacts self-management skills in some ways: understanding diabetes, recognizing symptoms, making informed choices, medication management, and preventing complications [4,5,6,7]. Additionally, education empowers patients to actively participate in their healthcare, fostering independence and improving outcomes. Continuous education and support are essential to ensure individuals have updated and relevant information to manage their diabetes efficiently [8]. Using a straightforward questionnaire to assess self-care is an effective way to gather reliable information [9].

Essential key points about its usefulness are as follows: (1) It identifies areas where patients need more education and support. (2) It explores psychosocial barriers like stress and social support, revealing why some people struggle with self-care. (3) Validated questionnaires ensure collected data are trustworthy for creating effective interventions. (4) They support research by providing measurable data to analyze patterns in self-care and blood sugar control. (5) Healthcare providers can tailor interventions to meet individual needs by evaluating self-care practices. (6) Repeated use of the questionnaire helps track changes in self-care and blood sugar control, providing feedback on intervention effectiveness [10].

Furthermore, a well-designed questionnaire supports research by providing measurable data to analyze self-care patterns, blood sugar control, and the effectiveness of interventions. It also provides insights into self-care practices and highlights psychosocial aspects that can hinder effective management, ultimately contributing to better diabetes outcomes [11].

Various instruments have been developed to assess the health-related quality of life (HRQOL) in diabetic patients, with the Diabetes Quality of Life (DQOL) questionnaire being one of the most widely used tools [12,13]. The original DQOL, introduced by the Diabetes Control and Complications Trial (DCCT) Research Group in 1988, comprises 46 core items organized into three primary domains: satisfaction with medical care, the impact of diabetes on daily life and emotional well-being, and concerns regarding diabetes-related complications. This instrument has been fundamental in understanding the psychosocial burden of diabetes and evaluating treatment effectiveness [13,14].

Over time, researchers have recognized the need for adaptations and modifications of the DQOL to ensure its applicability across different populations and healthcare settings. As a result, shorter versions have been developed to enhance usability in clinical practice while maintaining essential content. These adaptations provide a more efficient means of assessing patient-reported outcomes without compromising the reliability and validity of the instrument [15,16,17,18].

Despite the availability of validated tools, the continuous evolution of diabetes management and patient care necessitates the refinement or development of assessment instruments. Factors such as differences in study objectives, cultural and demographic variations, accessibility, and advancements in treatment strategies may require tailored measurement approaches. In some cases, existing instruments may not adequately capture specific aspects of patient experience, reinforcing the need for ongoing research in HRQOL assessment [19,20,21,22,23,24].

Existing diabetes assessment tools, such as DQOL and DSMQ, provide valuable insights but may not sufficiently capture the psychosocial and lifestyle barriers influencing self-care practices. For instance, while DQOL focuses on overall quality of life, it does not adequately assess social or emotional barriers. The DSMQ primarily evaluates self-management behaviors but lacks emphasis on interpersonal and psychological challenges. Given these limitations, our study aimed to develop a questionnaire addressing these gaps by assessing four key domains: physical activity, social relationships, emotional well-being, and dietary adherence.

## 2. Materials and Methods

This study presents a 23-item questionnaire designed to explore 4 critical domains influencing the lives of individuals with diabetes mellitus:

1. The role of physical activity in diabetes management.
2. The impact of diabetes mellitus on social relationships.
3. The emotional status of diabetic patients.
4. The role of diet in metabolic control.

### 2.1. Data Collection

Data were collected through SurveyMonkey, which allowed efficient and anonymous participation. However, we acknowledge that this method may introduce selection bias, as individuals without internet access or digital literacy may have been excluded. Future studies should consider mixed-method recruitment, including paper-based surveys, to enhance representativeness. Inclusion criteria required a clinically and paraclinically confirmed diagnosis of diabetes mellitus. Participants provided informed consent, ensuring confidentiality and anonymity. Data collection spanned five months (March 2024–July 2024).

### 2.2. Statistical Analysis

All data were analyzed using IBM SPSS Statistics (Version 20.0), with structural equation modeling (SEM) conducted via Amos (Version 20.0). Exploratory factor analysis (EFA) was used to identify latent constructs, employing Principal Axis Factoring with Varimax rotation. Confirmatory factor analysis (CFA) was conducted to validate the factor structure. Internal consistency was assessed using Cronbach’s Alpha.

As a first step, we conducted an exploratory factor analysis (EFA) to validate the developed instrument and measured variables. We used Principal Axis Factoring and Varimax rotation with Kaiser Normalization as the extraction method and confirmed the assumptions of linearity and correlation. All variables were considered in the analysis if they correlated at least 0.3. We assessed the Kaiser–Meyer–Olkin (KMO) measure for sampling adequacy and performed the Bartlett sphericity test. We considered KMO values greater than 0.5 and Bartlett test results with *p*-values less than 0.05 suitable for EFA. The initial questionnaire consisted of 29 items designed to evaluate the impact of diabetes on various aspects of patients’ lives, including physical, social, emotional, and dietary dimensions. The questionnaire was administered to a pilot sample of 35 individuals diagnosed with diabetes to assess its initial psychometric properties. These subjects responded to all 29 items, providing the data used for the initial stages of analysis.

After conducting the EFA, we removed variables that were not correlated with at least 0.3 to another. The final form of the questionnaire included 23 items (6 items containing sociodemographic information). The last form of the survey was administered, and confirmatory factor analysis (CFA) was performed using structural equation modeling (SEM).

The initial Kaiser–Meyer–Olkin (KMO) measure for sampling adequacy was below 0.70, indicating potential limitations in data suitability. To address this, we refined the questionnaire by removing poorly correlating items, which improved the KMO value to 0.763. Additionally, our confirmatory factor analysis (CFA) yielded a Tucker–Lewis Index (TLI) of 0.897, slightly below the 0.90 threshold. While this suggests room for improvement, other model fit indices (e.g., CFI = 0.915) indicated a reasonably strong model fit.

## 3. Results

A total of 150 respondents were included in the study, consisting of 88 men (58.67%) and 62 women (41.33%) (Table 1), for the final version of the questionnaire. The presence of a family history of diabetes was noted, with proportions being relatively equal: 63 respondents (42%) reported a family history of the disease, while 87 (58%) did not. The participants’ educational background was represented by 30 respondents (20%) having higher education, compared to 120 respondents (80%) with primary and secondary education.

Another factor considered was the duration of the disease, which was categorized as follows: 5 years, between 5 and 10 years, between 10 and 15 years, and between 15 and 20 years.

### 3.1. Exploratory Factor Analysis (EFA)

EFA (Table 2) was conducted on a pilot sample of 35 individuals. Initial reliability analysis yielded a Cronbach’s Alpha of 0.655, which improved to 0.755 after removing six poorly correlating items. The Kaiser–Meyer–Olkin (KMO) measure improved to 0.763, indicating suitability for factor analysis.

The factor extraction KMO measure of sampling adequacy was 0.696, below the desired threshold of 0.70, indicating potential issues with the adequacy of the data. After removing six poorly correlating items (questions 6, 7, 13, 16, 17, and 18), the KMO value improved to 0.763. Bartlett’s test of sphericity was significant (*p* < 0.001). Four factors were extracted (Table 2), which helped to define the factor structure better:

1. Physical Activity Management;
2. Social and Relational Impact;
3. Emotional Well-being;
4. Dietary Management.

### 3.2. Confirmatory Factor Analysis (CFA)

CFA was conducted on the entire sample (*n* = 150) to validate the four-factor model (Table 3). The CFA was used to test whether the data fit the hypothesized model of 4 latent constructs based on the retained 17 items.

The model fit indices indicated good fit:

The χ^2^ = 167.032, df = 113, *p* = 0.001, was significant, as expected, due to the sensitivity of chi-square to sample size; the minimum discrepancy CMIN/DF = 1.478 (acceptable range: 1–3). Additionally, other indexes suggested a good model fit: Comparative Fit Index (CFI) = 0.915 (good fit, >0.90); Tucker–Lewis Index (TLI) = 0.897 (close to the 0.90 threshold for a good fit); and the Incremental Fit Index (IFI) = 0.918 (confirming model adequacy).

The CFA results supported the four-factor structure, indicating that the refined 17-item questionnaire is a valid and reliable tool for assessing diabetes-related attitudes.

The final questionnaire consisted of 17 validated items across four domains. The chi-square test (χ^2^ = 167.032, df = 113, *p* = 0.001) was significant, likely due to sample size sensitivity rather than poor model fit. Future research should employ more extensive and diverse samples to validate the tool further.

### 3.3. Extracted Factors Analysis

Factor 1: Physical Activity Management: This factor includes items related to physical activity and its role in managing diabetes.

Factor 2: Social and Relational Impact: This factor includes items related to the social and relational burden of diabetes.

Factor 3: Emotional Well-being: This factor includes items related to the emotional impact of diabetes.

Factor 4: Dietary Management: This factor consists of items related to adherence to dietary recommendations and their role in blood sugar management.

**Figure 1:**
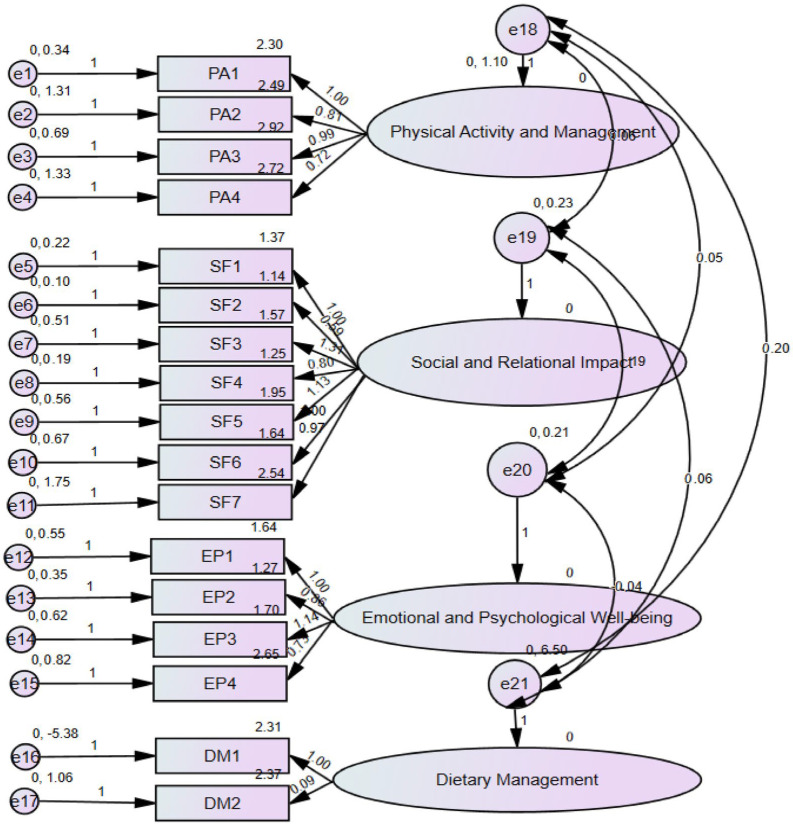
CFA with SEM validating questionnaire construct.

After the matrix rotation, the CFA in AMOS was performed to confirm the construct validity. Figure 1 illustrates the CFA model, displaying the four latent factors—physical activity management, social and relational impact, emotional well-being, and dietary management—and their corresponding 17 questionnaire items. An ellipse represents each factor, and the observed variables (items) are depicted as rectangles. Arrows from each factor to its respective items indicate the hypothesized relationships, with standardized factor loadings displayed on each path to show the strength and significance of these associations.

## 4. Discussion

Our study highlights the impact of lifestyle and psychosocial factors on diabetes management. Findings emphasize the importance of physical activity, social relationships, emotional well-being, and dietary practices in glycemic control. Our results demonstrate that satisfaction with physical activity levels and regular engagement in exercise are positively associated with better glycemic control. In contrast, avoidance or inconsistency in physical activity hinders effective diabetes management. These results align with previous studies demonstrating that sustained physical activity improves metabolic balance, social support influences self-management, and emotional distress affects treatment adherence [3,22,23,24,25,26]. The role of physical activity as a modifiable factor in diabetes self-care emphasizes the need for patient-centered interventions that promote adherence to exercise regimens.

The second key domain assessed was the social and relational impact of diabetes. Our study confirms that diabetes significantly affects interpersonal relationships, including those with spouses and family members, and contributes to perceived social burden. Participants frequently reported strained social interactions and reduced social satisfaction due to their condition. These findings align with existing research demonstrating that diabetes negatively impacts overall well-being and social functioning. Several population-based studies have noted that individuals with strong social support networks report higher health-related quality of life (HRQoL), particularly among married individuals, compared to unmarried counterparts [27,28]. Social isolation and stigma remain critical barriers to diabetes self-management, reinforcing the need for integrated psychosocial support in diabetes care strategies.

Another crucial dimension examined was emotional well-being, which was found to be significantly influenced by diabetes. Participants reported increased levels of emotional distress, anxiety, depression, sleep disturbances, and career limitations, all of which collectively contribute to a lower HRQoL. Psychological distress in diabetes has been widely documented, with studies in Saudi Arabia, Indonesia, India, and Nigeria confirming its negative impact on overall health outcomes [29,30,31,32]. These results highlight the necessity of incorporating mental health support into diabetes management programs, as addressing psychological burden may enhance treatment adherence and improve long-term health outcomes.

The final domain assessed in our study focused on dietary management. Proper adherence to dietary recommendations emerged as a fundamental component of diabetes self-care, significantly influencing blood glucose control and metabolic stability. Participants who adhered to prescribed dietary plans exhibited better glycemic regulation, consistent with previous studies from Ethiopia and Saudi Arabia [31]. Conversely, non-compliance with dietary recommendations was associated with metabolic imbalances and a heightened risk of diabetes-related complications. These findings reinforce the critical role of structured nutritional education and patient counseling in optimizing diabetes care.

Unlike DQOL and DSMQ, our questionnaire evaluates social burden, emotional distress, and perceived barriers to physical activity and diet. To strengthen validation, future studies should compare scores from our instrument with those from standard measures to assess concurrent validity.

Our study further identified several demographic and psychosocial factors influencing HRQoL in diabetic patients. Gender disparities were evident, with male participants reporting better HRQoL than female participants, consistent with previous findings [22,23,24]. The educational level also played a pivotal role, with higher education levels correlating with improved HRQoL, as also observed in several countries [25,27,28]. Additionally, both age and disease duration were significant predictors of HRQoL, as demonstrated in previous research [24,25,33]. Our study did not explicitly include socioeconomic status, comorbidities, and cultural background, which could influence diabetes management. These factors may significantly impact self-care behaviors, as individuals from lower socioeconomic backgrounds may have reduced access to healthcare resources. At the same time, cultural dietary practices can affect adherence to recommended nutrition plans. Future research should incorporate these demographic elements to understand how they shape diabetes self-management comprehensively. These results suggest that tailored interventions should consider demographic variations to optimize patient-centered care.

Despite the study’s strengths, several limitations should be noted. The reliance on self-reported data introduces potential response bias. The cross-sectional design prevents causal inference, and the homogeneous sample may limit generalizability. As a cross-sectional study relying on self-reported data, recall bias and social desirability bias are possible. To mitigate these limitations, future studies should triangulate self-reported data with objective measures (e.g., HbA1c levels) where possible. Future research should adopt longitudinal study designs to assess changes in HRQoL over time, incorporate diverse demographic groups to enhance external validity, and explore the dynamic interplay between the four identified domains. Additionally, qualitative research approaches, such as in-depth patient interviews, could provide a more nuanced understanding of the lived experiences of diabetic individuals, further informing tailored intervention strategies. Furthermore, our study presents only a single time-point assessment of patient attitudes. A longitudinal follow-up study would provide more comprehensive insights into the evolution of diabetes management behaviors over time and the impact of interventions on patient outcomes.

## 5. Conclusions

This study developed and validated a customized questionnaire assessing the attitudes of diabetic patients toward disease management. Findings underscore the importance of physical activity, social support, emotional well-being, and dietary adherence in improving HRQoL. Future interventions should prioritize self-management behaviors, reduce diabetes-related distress, and enhance family support systems to optimize patient outcomes.

## Figures and Tables

**Table 1:** Patient characteristics.

Characteristic	Respondents (n)	Percent (%)
History of Diabetes in Family		
Yes	63	42
No	87	58
Educational Level		
Primary school	63	42
High School	52	34.67
Faculty	35	23.22
Time since Diabetes diagnostic (years)		
0–5	27	18
5–10	54	36
10–15	34	22.87
15–20	35	23.33
Gender		
Male	88	58.67
Female	62	41.33

**Table 2:** EFA results (*n* = 35). Extraction method: Principal Component Analysis (rotation method: Equamax with Kaiser Normalization).

Item	Factor 1	Factor 2	Factor 3	Factor 4
Has your relationship with your spouse deteriorated due to diabetes?	0.722			
How satisfied are you with your social relationships or friendships?	0.683			
Is it a burden how others react to the fact that I have diabetes?	0.673			
I feel that I am less attractive to others because of my diabetes.	0.660			
How often do you feel physically ill?	0.608			
How often do you feel that diabetes limits your career?	0.590			
How satisfied are you with the time dedicated to physical activity?	0.546			
I avoid physical activity even though it improves my diabetes.		0.826		
I tend to skip planned physical activity.		0.792		
I engage in physical activities regularly to maintain my blood sugar levels.		0.760		
I am worried about my future health.		0.759		
Because of diabetes, I feel upset/depressed.			0.814	
How often do you sleep poorly/are stressed at night due to diabetes?			0.667	
I strictly adhere to the nutritional recommendations/diet provided by my doctor.			0.486	
The food I choose to eat helps me maintain my blood sugar levels.				0.841
Has your relationship with your spouse deteriorated due to diabetes?				0.840

Rotation converged in 4 iterations.

**Table 3:** CFA results (*n* = 150). Extraction method: Principal Axis Factoring (rotation method: Equamax with Kaiser Normalization).

Item	Factor 1	Factor 2	Factor 3	Factor 4	Cronbach Alpha
How satisfied are you with the time dedicated to physical activity?	0.813				0.797
I avoid physical activity even though it improves my diabetes.	0.709				
I engage in physical activities regularly to maintain my blood sugar levels.	0.692				
I tend to skip planned physical activity.	0.668				
Is it a burden how others react to the fact that I have diabetes?		0.621			0.719
Has your relationship with your spouse deteriorated due to diabetes?		0.616			
Is your condition—diabetes—a burden for your family?		0.595			
I feel that I am less attractive to others because of my diabetes.		0.484			
How often do you feel physically ill?		0.450			
How satisfied are you with your social relationships or friendships?		0.373			
Is it a burden to be mindful of what I eat?		0.233			
Because of diabetes, I feel upset/depressed.			0.510		0.669
How often do you feel that diabetes limits your career?			0.467		
How often do you sleep poorly/are stressed at night due to diabetes?			0.430		
I am worried about my future health.			0.279		
The food I choose to eat helps me maintain my blood sugar levels.				0.774	0.693
I strictly adhere to the nutritional recommendations/diet provided by my doctor.				0.677	

Rotation converged in 7 iterations.

## Data Availability

Data are available on request from the correspondence author.
